# SLC Transporter-Mediated Functional Imaging in Cancer Diagnosis

**DOI:** 10.3390/biom16071019

**Published:** 2026-07-12

**Authors:** Lumeng Zhang, Jun He

**Affiliations:** Department of Pathology and Genomic Medicine, Sidney Kimmel Medical College, Thomas Jefferson University, Philadelphia, PA 19107, USA

**Keywords:** solute carrier transporters (SLC), functional imaging, LAT1, GLUT, SLC2, SLC5, amino-acid PET tracers, metabolism-related molecular imaging

## Abstract

Functional imaging has become an important approach for evaluating tumor physiology in vivo beyond morphologic assessment. Radiotracers used for cancer imaging are designed to mimic endogenous substrates or substrate analogues, and their accumulation can depend on membrane transport, intracellular metabolism, and clearance from normal tissues. Specifically, SLC transporters contribute to tracer uptake and signal formation, linking transporter activity with measurable imaging signals in cancer. While [^18^F]FDG PET/CT remains the most widely used example of transporter-associated metabolic imaging, SLC transporter-mediated imaging strategies have been developed to assess amino acid transport, sodium-dependent glucose uptake, redox metabolism, lactate exchange, nucleoside metabolism, choline metabolism, and iodide accumulation. Herein, we provide an overview of current applications of SLC transporter-mediated functional imaging in cancer diagnosis, with an emphasis on glucose and amino acid systems. Special attention is given to the relationship between transporter physiology, tracer uptake, tumor type, and diagnostic application.

## 1. Introduction

Molecular and functional imaging have become important tools for cancer diagnosis and clinical management. Unlike anatomic imaging, which mainly reflects tumor size, location, and morphology, functional imaging can detect physiological processes in vivo [1]. These processes include nutrient uptake, membrane transport, proliferation, hypoxia, redox status, and receptor expression [1,2,3]. Positron emission tomography (PET), single-photon emission computed tomography (SPECT), and selected magnetic resonance imaging (MRI) methods therefore provide information that cannot be obtained from structural imaging alone [2,3]. Cancer cells often reprogram their metabolism to support growth, survival, and adaptation to stress [4,5,6,7]. One common feature of tumor reprogramming is increased uptake of nutrients and other solutes, including glucose, amino acids, lactate, nucleosides, choline, and metal ions [7]. These metabolic changes provide useful targets for functional imaging. The most widely used example is 2-deoxy-2-[^18^F] fluoro-D-glucose ([^18^F]FDG) PET/CT, which images glucose utilization and is routinely employed for tumor detection, staging, restaging, and treatment response assessment [3]. Nevertheless, modern oncologic PET imaging extends beyond [^18^F]FDG. Numerous radiotracers have been developed to visualize key features of tumor biology, including amino acid transport, nucleotide metabolism, choline metabolism, receptor expression, hypoxia, and other tumor-associated pathways [3]. Many of these imaging signals depend on membrane transport. The solute carrier (SLC) superfamily comprises numerous membrane transporters that mediate the movement of amino acids, sugars, nucleosides, ions, and other solutes across cellular membranes [8,9,10]. These transporters regulate biosynthesis, redox balance, nutrient sensing, and growth signaling in cancer cells [11,12]. SLC transporters connect extracellular nutrient availability with intracellular metabolism and can strongly influence the uptake of imaging tracers.

While SLC transporters are central to nutrient uptake and metabolite exchange, their role in cancer diagnostic imaging remains unevenly explored [3,13,14]. This gap is critical, because many functional imaging tracers depend on transporter-mediated cellular entry. Herein, we provide an overview of SLC transporter-mediated functional imaging in cancer diagnosis, with emphasis on glucose transporters and amino acid transporters.

## 2. The SLC Transporter Superfamily and Its Relevance to Cancer Functional Imaging

The SLC superfamily, one of the largest groups of membrane transport proteins in the human genome, consists of 65 families with more than 450 transporters [8]. SLC transporters are localized to both in the plasma membrane and intracellular membranes [8,12]. Their substrates include sugars, amino acids, nucleosides, monocarboxylates, organic ions, metals, and many other solutes [8,15]. SLC transporters regulate the exchange of these nutrients and metabolites between the extracellular space, cytosol, and intracellular organelles [16,17]. Tumor cells depend on extracellular nutrients to sustain metabolism and growth, especially through increased glucose and amino acid uptake [7]. Notably, many SLC transporters are aberrantly expressed in cancer, allowing tumor cells to acquire nutrients from the extracellular environment [1].

Solute movement across membranes is controlled by several classes of membrane protein [16,17,18,19]. ATP-powered pumps use ATP hydrolysis to build and maintain electrochemical gradients [16,18]. Ion channels form pores that allow rapid ion flow down electrochemical gradients [16]. Transporters, also called carriers, bind specific substrates and move them across membranes by facilitated diffusion or by coupling transport to ion gradients [16,17]. Most transporters do not form continuously open pores. Instead, they usually work through an alternating-access mechanism [20,21]. In this model, substrate binding is coupled to conformational changes that expose the binding site to one side of the membrane and then to the other [20,21]. This selectivity is also relevant to functional imaging, as many radiotracers are designed to resemble endogenous substrates and enter cells through SLC-mediated transport [13,16,17]. Many functional imaging tracers are polar substrate analogs that cannot freely cross the lipid bilayer and require transporter-mediated cellular entry [22]. Their accumulation in tumor tissue often depends on transporter-mediated cellular entry, intracellular metabolism, retention, and clearance from normal tissues [13]. SLC uptake transporters deserve attention in functional imaging because they can concentrate endogenous nutrients and metabolites into cells. When a transporter is enriched in a specific tumor type, tissue compartment, or metabolic state, a radiolabeled substrate or substrate analog can be used to visualize transporter-associated uptake in vivo [13].

Several established cancer imaging methods are linked to the SLC transporter function. Representative SLC transporter families, related substrates, and imaging tracers are summarized in Table 1. FDG uptake is closely related to glucose transport through the glucose transporter (GLUT) family, especially GLUT1 (SLC2A1), although hexokinase activity, tumor cellularity, hypoxia, and inflammation also affect FDG signal [23,24]. Sodium-dependent glucose transporters, including SGLT1 (SLC5A1) and SGLT2 (SLC5A2), can be imaged with α-methyl-4-deoxy-4-[^18^F]fluoro-D-glucopyranoside ([^18^F]Me4FDG) PET approaches [25,26]. Amino acid PET tracers, such as L-[methyl-^11^C]methionine ([^11^C]MET), O-(2-[^18^F]fluoroethyl)-L-tyrosine ([^18^F]FET), 6-[^18^F]fluoro-L-3,4-dihydroxyphenylalanine ([^18^F]FDOPA), 3-[^18^F]fluoro-α-methyl-L-tyrosine ([^18^F]FAMT), and 4-borono-2-[^18^F]fluoro-L-phenylalanine ([^18^F]FBPA), are associated with amino acid transport systems, especially L-type amino acid transporter 1 (LAT1 (SLC7A5)) in various tumors [27,28,29,30,31,32]. Other examples include 4-[^18^F]fluoroglutamine ([^18^F]FGln) for glutamine transport and (4S)-4-(3-[^18^F]fluoropropyl)-L-glutamate ([^18^F]FSPG) for system xC^−^/xCT activity (SLC7A11, with SLC3A2 as the heavy-chain partner) [33]. Lactate-related tracers, such as 3-[^18^F]fluoro-2-hydroxypropionate ([^18^F]FLac), have also been developed to assess MCT-mediated lactate transport, including MCT1 (SLC16A1) and MCT4 (SLC16A3) [34].

## 3. SLC Glucose Transporters in Functional Imaging

### 3.1. Molecular and Physiological Characteristics of SLC Glucose Transporters

Altered glucose utilization is one of the most recognized metabolic features of cancer, which provides the physiological basis for glucose-based PET imaging [61,62]. Two major SLC families mediate glucose transport. The SLC2 family, also known as the GLUT family, mediates sodium-independent facilitated glucose transport [23,24]. The SLC5 family, also known as the SGLT family, mediates sodium-dependent glucose co-transport [25,26]. While GLUT-mediated glucose transport provides the biological basis for routine ^18^F-FDG PET/CT, SGLT-mediated glucose uptake has driven the development of newer tracers such as ^18^F-Me4FDG [23,38].

The SLC2 family comprises facilitative glucose transporters that move glucose and related hexoses across biological membranes without direct sodium coupling [63]. These transporters generally move glucose down its concentration gradient [63]. Among this family, GLUT1 and GLUT3 are the most widely utilized for cancer imaging [23]. GLUT1 is encoded by *SLC2A1* gene and supports basal glucose uptake in many normal tissues [63]. In tumors, GLUT1 is frequently upregulated to support increased glucose demand in response to rapid growth, hypoxia, and metabolic stress [23]. GLUT3, encoded by SLC2A3, has a high affinity for glucose and may help tumor cells maintain glucose uptake under nutrient-limited conditions [23,63]. ^18^F-FDG enters cells mainly through GLUT transporters and then is phosphorylated by hexokinase to ^18^F-FDG-6-phosphate [64]. Unlike glucose-6-phosphate, ^18^F-FDG-6-phosphate is poorly metabolized further and is retained inside the cell [64]. Therefore, FDG PET signal reflects both glucose transport and intracellular phosphorylation. GLUT-related imaging with ^18^F-FDG PET/CT is well established and widely used in oncology [65,66,67]. Although GLUT1 expression is often associated with FDG uptake, FDG PET/CT should not be considered as a direct measurement of GLUT1 alone. Meyer et al. reported that while GLUT1 expression showed only a moderate overall association with FDG-PET-derived SUV values, GLUT3 expression showed a weaker correlation with FDG-PET-derived SUV values [23]. Collectively, these findings underscore that tumor FDG uptake is regulated by a multifactorial process rather than by GLUT expression alone. FDG uptake can also be influenced by hexokinase enzyme activity, tumor cellularity, blood flow, hypoxia, viable tumor fraction, and inflammatory cell infiltration [24].

The SLC5 family has a different transport mechanism. SGLT proteins use the sodium gradient across the plasma membrane to drive secondary active glucose uptake, allowing cells to accumulate glucose against its concentration gradient [38,68]. SGLT1 is encoded by SLC5A1 and is classically involved in intestinal glucose absorption [69]. SGLT2 is encoded by SLC5A2 and is mainly expressed in the proximal renal tubule, where it mediates most renal glucose reabsorption [69]. In cancer, SGLTs have received increasing attention because some tumors may use sodium-dependent glucose uptake in addition to GLUT-mediated uptake [26,38]. As ^18^F-FDG mainly reflects GLUT-associated uptake, it is not an ideal tracer for SGLT activity. Therefore, FDG PET may not fully capture SGLT-dependent glucose transport in tumors. This limitation has encouraged the development of SGLT-targeted PET tracers. One representative tracer is ^18^F-Me4FDG, which, unlike ^18^F-FDG, is transported by SGLTs rather than GLUTs [25]. ^18^F-Me4FDG provides a more specific imaging approach for sodium-dependent glucose uptake [38].

### 3.2. Clinical and Translational Applications of SLC Glucose Transport Imaging

#### 3.2.1. GLUT-Mediated Imaging with ^18^F-FDG PET/CT

^18^F-FDG PET/CT is widely used for tumor diagnosis, staging, restaging, treatment response assessment, radiotherapy planning, and prognostic evaluation [28]. Its clinical applications include lymphoma, lung cancer, breast cancer, colorectal cancer, pancreatic cancer, and melanoma [3,28,35,36,37]. The strength of FDG PET/CT is its ability to show increased glucose use across the whole body [28]. Tumors with high glucose transport and high hexokinase activity usually show increased FDG retention [23,28]. This makes the FDG PET/CT approach useful for detecting metabolically active lesions and distant metastases [35,70], and allows clinicians to compare metabolic tumor burden before and after therapy [3,37]. In lung cancer, FDG PET/CT is crucial for staging and treatment planning [35]. FDG uptake in lung cancer has been associated with GLUT1 expression, hexokinase II expression, and tumor proliferation markers such as proliferating cell nuclear antigen (PCNA) [24]. FDG uptake varies by histological subtypes, with squamous cell carcinoma often showing stronger FDG uptake and higher GLUT1 expression than adenocarcinomas [24,70]. Some well-differentiated adenocarcinomas, especially early lesions, may show relatively low FDG uptake [70]. In breast cancer, FDG uptake is often higher in biologically aggressive molecular subtypes [70,71]. Triple-negative and HER2-positive tumors generally exhibit higher FDG avidity than hormone receptor-positive tumors [71]. FDG PET/CT is more effectively utilized for advanced breast cancer, recurrent disease, and treatment response assessment than for the routine screening of small, early-stage tumors [71]. In colorectal cancer, FDG PET/CT is commonly used to evaluate recurrence and metastatic disease [70]. It can help clarify equivocal findings on CT or MRI and identify metabolically active lesions in the liver, lymph nodes, peritoneum, or distant organs [36]. Notably, FDG uptake in colorectal cancer can be affected by lesion size, mucinous histology, inflammation, and tumor heterogeneity [36,72]. For this reason, FDG PET/CT should be interpreted together with anatomical imaging and clinical information. In pancreatic cancer, FDG PET/CT aids in identifying metabolically active primary tumors and distant metastases, while potentially provides prognostic information in selected patients [73]. A major limitation, however, is that pancreatic inflammation can also show increased FDG uptake, as both pancreatitis and pancreatic cancer can be FDG-avid [72,73]. Therefore, FDG positivity in the pancreas is not tumor-specific and must be interpreted alongside CT, MRI, clinical findings, and pathology.

Despite its broad clinical values, GLUT-mediated FDG PET/CT has several important limitations. Firstly, GLUT-mediated glucose uptake is not specific to malignant cells [72]. Secondly, activated immune cells, infection, inflammation, brown adipose tissue, wound healing, and physiologically glucose-avid organs can all show increased FDG uptake [72]. In addition, the brain and myocardium typically exhibit high physiological glucose consumption, which can reduce tumor contrast in selected settings [67]. FDG PET/CT is a powerful glucose metabolism imaging method, but it is not a cancer-specific or GLUT-specific imaging method [3].

#### 3.2.2. SGLT-Mediated Imaging with ^18^F-Me4FDG

SGLT-based strategies represent a novel frontier in glucose transporter imaging [38]. It evaluates sodium-dependent glucose uptake rather than the classical GLUT-dependent pathway [26]. This distinction is critical, as certain tumors express SGLT1 or SGLT2 and may leverage these pathways for glucose utilization [38]. ^18^F-Me4FDG, the best-studied SGLT PET tracer, was designed to be transported by SGLTs rather than GLUTs, which allows more direct imaging of SGLT activity than conventional ^18^F-FDG [38]. Scafoglio et al. demonstrated functional SGLT expression in human pancreatic and prostate cancers [26]. Their preclinical studies using human tumor specimens and xenograft models of pancreatic and prostate cancer exhibited uptake of SGLT-targeted glucose tracers, which was subsequently reduced by SGLT2 inhibitors, confirming the specificity of this pathway. SGLT imaging may be useful not only for tumor detection, but also for selecting tumors that could be sensitive to SGLT-targeted therapy. One important example is early lung adenocarcinoma, in which SGLT2 expression has been reported in premalignant lesions and well-differentiated adenocarcinomas [26]. Beyond lung cancer, SGLT imaging has also been explored in brain tumors; in this context, it offers a distinct advantage over conventional FDG PET, as the high physiological FDG uptake of normal brain tissue often obscuring tumors is minimized with SGLT-targeted tracers [74]. Kepe et al. reported Me-4FDG uptake in patients with WHO grade III or IV astrocytomas and linked this uptake to SGLT2 expression in neoplastic glioblastoma cells and proliferating tumor microvasculature [75]. Early human biodistribution work suggests that ^18^F-Me4FDG can be administered safely and has favorable biodistribution for PET imaging [76]. However, more studies are still needed to define its tumor specificity, reproducibility, clinical indications, and ability to predict response to SGLT2 inhibitors. Nonetheless, ^18^F-Me4FDG may ultimately provide complementary information to FDG PET.

## 4. SLC Amino Acid Transporters in Functional Imaging

### 4.1. Molecular and Physiological Characteristics of SLC Amino Acid Transporters

Unlike glucose uptake, amino acid uptake is not controlled by one dominant transporter family but by overlapping transport systems with distinct substrate preferences, driving forces, and exchange mechanisms [77]. Within this framework, SLC amino acid transporters coordinate cellular uptake and exchange to sustain the metabolic needs of tumor cells, ensuring their survival, proliferation, and adaptation to the microenvironment [78]. Among these, System L is particularly highly relevant for cancer imaging due to its sodium-independent transport of large neutral amino acids, including branched-chain and aromatic amino acids [79]. Its primary member, LAT1, typically functions as a heterodimer with the heavy chain SLC3A2 (CD98hc), which is required for membrane localization and transporter stability [80]. Mechanistically, LAT1 operates as an obligate exchanger that utilizes intracellular amino acids such as glutamine to drive the influx of extracellular essential amino acids [81]. This exchange is actively sustained by ASCT2 and SLC38 (SNAT) transporters, which maintain the necessary intracellular pools of neutral amino acids and glutamine [82]. Together, this coupled transport network fuels protein synthesis, mTORC1 signaling, and tumor growth [83]. SLC38A9 adds another layer to this network at the lysosomal membrane. It functions in the Rag–Ragulator–mTORC1 amino acid sensing pathway and promotes the efflux of essential amino acids, including leucine, from lysosomes after lysosomal protein degradation. In this way, SLC38A9 links lysosomal amino acid recycling with cytosolic amino acid availability and mTORC1 activation [84].

Other amino acid transporters reflect different aspects of tumor physiology. For example, SLC7A11 (xCT) imports cystine in exchange for glutamate and supports glutathione synthesis and ferroptosis resistance, whereas SLC6A14 (ATB^0,+^) has broad substrate specificity and can concentrate many neutral and cationic amino acids [85,86]. In summary, the amino-acid based imaging signals reflect more than nutrient uptake; they are shaped by transporter activity, substrate competition, intracellular amino acid exchange, redox state, metabolic stress, and physiological background uptake in normal tissues [77].

### 4.2. Diagnostic Functional PET Imaging of Amino Acid Transport

SLC-driven amino acid transport programs can be measured in vivo by PET imaging [87]. Amino acid tracer PET provides a functional readout of tumor amino acid transport rather than only tumor anatomy [31,87]. It is attractive in oncology because amino acids support both biomass production and nutrient signaling, and tumors often increase amino acid uptake under growth and microenvironmental stress [88]. Several clinically used amino acid PET tracers act as functional probes of transmembrane amino acid transport, with uptake largely reflecting increased transport capacity rather than direct incorporation into proteins [31,89,90]. For tyrosine-based tracers such as O-(2-[^18^F]fluoroethyl)-L-tyrosine (^18^F-FET), uptake is mainly linked to system L transport activity [31]. ^18^F-FDOPA (6-[^18^F] fluoro- L-3,4-dihydroxyphenylalanine) is also a large neutral amino acid analogue that enters the brain and many tumors predominantly via system L transporters (LAT1 and LAT2) [27,91]. While ^18^F-FDG (Fluorodeoxyglucose) PET remains the most widely used oncologic PET approach, comparisons between FDG and amino acid PET tracers are clinically important [28,92]. FDG primarily reflects glucose uptake and glycolytic activity; however, its tumor specificity can be limited by high physiological uptake in the brain and nonspecific accumulation in inflammatory tissues [72,93]. Amino acid PET tracers can help overcome these limitations in selected settings, particularly in neuro-oncology [94,95]. For example, ^18^F-FET has shown low uptake in inflammatory cells and inflammatory lymph nodes in experimental and comparative studies [31]. This property may help distinguish tumor uptake from inflammatory uptake in some clinical situations. Accordingly, amino acid PET is now used in neuro-oncology, and the joint EANM/EANO/RANO/SNMMI procedure guidelines include radiolabeled amino acids such as MET, FET, and FDOPA, together with FDG, for glioma PET imaging [28]. These amino acid PET tracers can be interpreted as functional readouts of tumor amino acid transport, especially LAT-linked SLC transport pathways (Table 2). Amino acid PET can also provide information relevant to therapy planning. A clear example is L-boronophenylalanine (BPA)-based boron neutron capture therapy (BNCT). BPA is an amino acid-derived boron delivery agent whose uptake can be mediated by LAT1, LAT2, and ATB^0,+^ [96]. The PET analogue ^18^F-FBPA has been used to estimate BPA-derived boron accumulation in tumors and normal tissues prior to BNCT. This example illustrates how amino acid transporter-linked PET can help guide the delivery of amino acid-mimicking therapeutics, although BPA should be considered transporter-linked rather than LAT1-specific [96,97,98]. Most amino acid PET studies in clinical use are linked to LAT-mediated transport, but several other amino acid transporters have also been investigated in cancer imaging. For example, 4-[^18^F]fluoroglutamine PET has been evaluated as a non-invasive marker of ASCT2 expression in lung cancer models, and [^18^F]fluciclovine uptake has been associated with ASCT2 and LAT1 activity in prostate cancer and glioma studies [47,48,49]. ATB^0,+^ is another broad-specificity amino acid transporter of interest, but its imaging applications remain mainly preclinical. Chiotellis et al. first reported the synthesis and preliminary biological evaluation of is O-2-((2-[^18^F]fluoroethyl)methylamino)ethyl-L-tyrosine ([^18^F]FEMAET), a cationic amino acid analogue developed to probe ATB^0,+^ transport activity [44]. These examples suggest that amino acid PET may extend beyond LAT-centered imaging, although most non-LAT transporter-linked tracers still require further validation before routine oncologic use.

Following functional uptake imaging, a complementary question is whether the transporter protein itself can be visualized [100]. Amino acid PET tracers are valuable because they measure transport phenotypes in vivo; however, tracer uptake should not be interpreted as a direct measure of transporter protein abundance [14,101]. Substrate-analog PET, including FET, MET, FDOPA, FAMT, and related tracers, can identify active amino acid import phenotypes and support neuro-oncology tasks such as lesion detection, tumor delineation, and response assessment [28,31,32,42,43]. Target-directed imaging instead aims to visualize the transporter protein on tumor cells and may be particularly useful when tracer uptake does not correlate with transporter expression [100]. One example is immuno-PET of LAT1, which uses monoclonal antibodies targeting the extracellular domain of native human LAT1. Ikotun and colleagues radiolabeled an anti-LAT1 monoclonal antibody with ^89^Zr and achieved clear visualization of HCT116 colorectal cancer xenografts [100]. Importantly, tumor accumulation was reduced by approximately 55% following blockade with excess unlabeled antibody, supporting target-specific binding. This study established the feasibility of imaging a specific amino acid transporter target and also illustrates the rationale underlying immuno-PET: antibodies can provide high specificity and affinity for cell-surface antigens, while the long half-life of ^89^Zr is well suited to antibody pharmacokinetics [100,102]. Recent studies have also developed new LAT1-targeted PET probes based on small-molecule amino acid analogues. A recent review summarized the development of LAT1-specific PET radiotracers for improving tumor specificity, reducing inflammation-related false-positive uptake, and supporting lesion delineation and prognostic assessment [103]. In addition, Kondo et al. reported a LAT1-selective PET tracer, 5-[^18^F]F-αMe-3BPA, as a companion imaging agent for a structurally matched boron-containing analog used in boron neutron capture therapy [104]. These studies show that LAT1 remains an active target for PET probe development. Most LAT1-targeted amino acid tracers are still small-molecule substrates or analogues. Their uptake can be affected not only by LAT1 expression, but also by transport activity, blood supply, and tracer washout. Therefore, antibody-based LAT1 immuno-PET may provide complementary information by showing whether LAT1 protein is present on the tumor cell surface.

### 4.3. LAT1-Targeted Optical and Nanoparticle Imaging: NIR Probes and Nanomaterial Platforms

Importantly, LAT1-targeted imaging is not limited to PET. NIR fluorescence probes and nanomaterials provide complementary approaches for real-time, high-resolution imaging at tissue scale, making them particularly attractive for ex vivo assessment, fluorescence-guided surgery, and intraoperative navigation rather than whole-body staging [105,106]. Optical probes may help surgeons visualize tumor margins, identify residual tumor tissue, and guide local resection decisions in real time [106]. A recent study described an LAT1-specific NIR fluorescent probe for cancer-targeted imaging, demonstrating LAT1 overexpression in triple-negative breast cancer models and enabling both in vitro and in vivo fluorescence visualization using an LAT1-targeted NIR dye conjugate [107]. In parallel, LAT1-targeted nanoprobe development has explored near-infrared-emitting gold nanoclusters functionalized with amino acid motifs to recognize LAT1-high cells [108]. For example, methionine-functionalized gold nanoclusters (Met-NHs-AuNCs) have been reported to label LAT1-overexpressing cancer cells in fluorescence imaging experiments, supported by complementary binding and docking analyses [108]. Additional study has similarly described ultra-bright, water-soluble gold nanoclusters for LAT1-targeted cancer cell imaging [109]. These optical and nanoparticle-based strategies are not intended to replace amino acid tracer PET. Rather, they expand the LAT1 imaging toolkit by providing real-time, tissue-scale imaging capabilities that can complement the whole-body sensitivity of PET and may be particularly useful for local tumor visualization during image-guided procedures. Nevertheless, optical imaging approaches have important limitations: restricted tissue penetration depth, autofluorescence-related signal interference, and lower standardization compared with PET can all reduce sensitivity and clinical applicability [110].

Taken together, these imaging modalities provide different information on the SLC transporter function. While amino acid tracer PET is the most widely studied method for imaging transport activity in vivo, newer LAT1 immuno-PET probes may allow more direct evaluation of LAT1 expression in tumors. Optical probes, including near-infrared dyes and nanoparticle-based agents, can provide rapid and high-resolution signals at the tissue level, although their tissue penetration is more limited than PET. Thus, PET and optical imaging may have complementary roles in SLC-based tumor imaging, with PET suitable for whole-body evaluation and optical approaches useful for local or tissue-level.

The deregulation of SLC transporters in cancers enables functional imaging of tumors. Table 3 shows the upregulation of these transporters in a variety of cancers.

## 5. Conclusions

SLC transporter-mediated imaging is rapidly emerging as a cornerstone of functional cancer imaging. Because SLC transporters provide entry routes for many nutrients, metabolites, and imaging probes, their altered expression and activity are intrinsically linked to tumor metabolism. While [^18^F]FDG PET/CT remains the gold standard—reflecting glucose transporter-associated uptake combined with intracellular metabolic trapping—amino acid tracer PET is also well established. Particularly in brain tumors, amino acid PET successfully captures transport activity through LAT-linked and other SLC-associated systems. However, amino acid PET signals should not be interpreted as equivalent across tracers. ^11^C-MET is a natural amino acid tracer, and its tumor signal can reflect both transporter-mediated uptake and downstream methionine use, including protein incorporation. In contrast, synthetic amino acid analogues such as ^18^F-FET and ^18^F-FAMT are transported into tumor cells but are not substantially incorporated into proteins, so their tumor signals are more closely related to transporter-mediated uptake than to protein synthesis. ^18^F-FDOPA also depends on LAT1/LAT2-mediated uptake, but AADC-related intracellular metabolism can further influence tracer retention in selected tumors, especially neuroendocrine or catecholaminergic tumors. Recent studies have further expanded this landscape beyond traditional FDG and classical amino acid PET. SGLT-targeted glucose tracers, ASCT2- and xCT-related amino acid probes, MCT-linked lactate tracers, nucleoside and choline tracers, NIS-mediated radioiodine imaging, OATP-linked hepatobiliary MRI, and copper-based PET all demonstrate that distinct SLC systems can provide unique insights into tumor physiology.

Future studies must place greater emphasis on how individual SLC transporters shape tracer kinetics across different tumor types. This granular understanding will clarify why some tumors exhibit robust uptake of a given tracer while others do not. Furthermore, a deeper knowledge of transporter expression profiles, substrate preferences, and tissue backgrounds will guide the design of more specific imaging probes. Ultimately, as this field develops, SLC-based imaging will play a pivotal role in refining tumor characterization, guiding tracer selection, and uncovering novel metabolic signatures for precision oncology.

## Figures and Tables

**Table 1:** SLC transporter families related to diagnostic functional imaging in cancer.

Substrate/Functional Category	SLC Subfamily/Representative Genes	Common Name	Representative Imaging Tracer
Glucose transport	SLC2 family; mainly SLC2A1, also SLC2A3	GLUT1, GLUT3	[^18^F]FDG PET/CT [3,28,35,36,37]
Sodium-dependent glucose transport	SLC5 family; SLC5A1, SLC5A2	SGLT1, SGLT2	Me4FDG PET/Me-4FDG PET, SGLT-targeted PET [38]
Iodide transport	SLC5 family; SLC5A5	NIS, sodium/iodide symporter	^123^I scintigraphy/SPECT, ^124^I PET/CT, ^131^I whole-body scan, ^99m^Tc-pertechnetate imaging [39,40,41]
Large neutral amino acid transport	SLC7 family; SLC7A5 with SLC3A2	LAT1, system L	[^11^C]MET PET, [^18^F]FET PET, [^18^F]FDOPA PET, [^18^F]FAMT PET, [^18^F]FBPA PET, LAT1 immunoPET [28,30,31,32,42,43]
Broad neutral/cationic amino acid transport	SLC6 family; SLC6A14	ATB^0,+^	ATB^0,+^-selective amino acid PET tracer, such as cationic O-2-[^18^F]fluoroethyl-L-tyrosine analogues, mainly preclinical [44]
System A amino acid transport	SLC38 family; mainly SLC38A1, SLC38A2	SNAT1, SNAT2; system A	[^11^C]MeAIB PET, [^11^C]AIB PET, [^18^F]FAMP PET, [^18^F]MeFAMP PET, mainly preclinical [45,46]
Glutamine/neutral amino acid transport	SLC1 family; SLC1A5	ASCT2	[^18^F]fluoroglutamine PET [47,48,49]
Cystine/glutamate exchange and redox imaging	SLC7 family; SLC7A11 with SLC3A2	xCT, system xC^-^	[^18^F]FSPG PET [50]
Lactate/monocarboxylate transport	SLC16 family; mainly SLC16A1, also SLC16A3	MCT1, MCT4	[^18^F]FLac PET, [^18^F]FACH PET, MCT-targeted PET, mainly preclinical [34,51,52]
Choline transport/membrane synthesis imaging	SLC44 family; mainly SLC44A1	CTL1, choline transporter-like protein 1	[^11^C]choline PET/CT, [^18^F]fluorocholine PET/CT [53,54,55,56]
Organic anion transport/hepatobiliary MRI	SLCO family, historically OATP/SLC21; SLCO1B1, SLCO1B3	OATP1B1, OATP1B3	Gadoxetic acid-enhanced MRI, Gd-EOB-DTPA MRI, EOB-MRI [57,58]
Copper transport/metal-ion imaging	SLC31 family; SLC31A1	CTR1, copper transporter 1	^64^CuCl_2_ PET/CT, preclinical and early clinical [59,60]

**Table 2:** Amino acid PET tracers as functional readouts of LAT transport programs in cancer.

Tracer	Mechanistic Driver(s) of Uptake (SLC Link)	Clinical Applications	Advantages	References
^18^F-FET O-(2-[^18^F]fluoroethyl)-L-tyrosine	System L/LAT-linked uptake, not incorporated into proteins.	Glioma imaging; recurrence vs. treatment effect; selected extracranial tumors.	Low brain background; less inflammatory uptake.	[28,31,32]
^11^C-MET L-[methyl-^11^C]methionine	Predominantly system L (LAT-linked); uptake correlates with LAT1/4F2hc in glioma cohorts.	Glioma delineation and recurrence assessment.	High brain tumor contrast; useful for delineation.	[28,30]
^18^F-FDOPA 6-[^18^F]fluoro-L-3,4-dihydroxyphenylalanine	System L (LAT1/LAT2) uptake; AADC-related handling may influence signal.	Glioma grading/recurrence; neuroendocrine and catecholaminergic tumors.	Low brain background; good lesion conspicuity.	[27,28,29]
^18^F-FAMT 3-[^18^F]fluoro-α-methyl-L-tyrosine	LAT1-associated.	Oral squamous cell carcinoma imaging; potential companion-style imaging for LAT1-targeted therapy.	Higher LAT1 specificity and tumor specificity; lower uptake in inflammatory or granulomatous lesions, which may reduce false-positive inflammation-related signal.	[42,43,99]

**Table 3:** Upregulated SLC transporters in cancerous versus normal tissues.

SLC Transporter	Cancer Types	References
GLUT1	Elevated expressions observed in many cancers including breast, ovarian, colorectal, lung, cervical, thyroid, hepatic, pancreatic, brain, and oral squamous carcinoma etc.	[111,112,113,114,115,116,117]
GLUT3	thyroid cancer, breast cancer, lung cancer, colorectal cancer	[116,118,119,120]
SGLT1	pancreatic cancer, ovarian cancer	[121,122]
LAT1	Elevated expressions observed in many cancers including lung, prostate, liver, melanoma, brain, breast, gastric, and esophageal cancer etc.	[79]
ATB^0,+^	colorectal cancer, lung cancer, pancreatic cancer, hepatocarcinoma	[123,124,125,126]
SNAT1	melanoma, breast cancer, ovarian cancer, osteosarcoma	[127,128,129,130,131]
SNAT2	breast cancer	[132]
ASCT2	Elevated expressions observed in many cancers including hepatocellular carcinoma, lung cancer, colorectal cancer, gastric cancer, clear cell renal cell carcinoma, ovarian cancer, and breast cancer etc.	[133]
xCT	lung cancer, hepatocellular carcinoma, melanoma, lymphoma, ovarian cancer, breast cancer, glioma, prostate cancer	[50,134,135,136]
MCT1	Elevated expressions observed in many cancers including head and neck cancer, breast cancer, colon cancer, lung cancer, bladder cancer and glioblastoma	[137]
MCT4	glioma, breast cancer, gastric cancer	[138,139,140,141]
CTL1	pancreatic ductal adenocarcinoma, tongue squamous cell carcinoma	[142,143]
CTR1	breast cancer, lung cancer, melanoma	[144,145,146]

## Data Availability

No new data were created or analyzed in this study.

## References

[1] Nwosu Z.C., Song M.G., di Magliano M.P., Lyssiotis C.A., Kim S.E. (2023). Nutrient transporters: Connecting cancer metabolism to therapeutic opportunities. Oncogene.

[2] Lewis D.Y., Soloviev D., Brindle K.M. (2015). Imaging tumor metabolism using positron emission tomography. Cancer J..

[3] Farwell M.D., Pryma D.A., Mankoff D.A. (2014). PET/CT imaging in cancer: Current applications and future directions. Cancer.

[4] Faubert B., Solmonson A., DeBerardinis R.J. (2020). Metabolic reprogramming and cancer progression. Science.

[5] Wise D.R., Thompson C.B. (2010). Glutamine addiction: A new therapeutic target in cancer. Trends Biochem. Sci..

[6] Lin D.-W., Carranza F.G., Borrego S., Lauinger L., Dantas de Paula L., Pulipelli H.R., Andronicos A., Hertel K.J., Kaiser P. (2025). Nutrient control of splice site selection contributes to methionine addiction of cancer. Mol. Metab..

[7] Pavlova N.N., Thompson C.B. (2016). The emerging hallmarks of cancer metabolism. Cell Metab..

[8] Pizzagalli M.D., Bensimon A., Superti-Furga G. (2021). A guide to plasma membrane solute carrier proteins. FEBS J..

[9] Hediger M.A., Clémençon B., Burrier R.E., Bruford E.A. (2013). The ABCs of membrane transporters in health and disease (SLC series): Introduction. Mol. Asp. Med..

[10] Lin L., Yee S.W., Kim R.B., Giacomini K.M. (2015). SLC transporters as therapeutic targets: Emerging opportunities. Nat. Rev. Drug Discov..

[11] Bhutia Y.D., Babu E., Ramachandran S., Ganapathy V. (2015). Amino acid transporters in cancer and their relevance to “glutamine addiction”: Novel targets for the design of a new class of anticancer drugs. Cancer Res..

[12] Bröer S. (2020). Amino acid transporters as targets for cancer therapy: Why, where, when, and how. Int. J. Mol. Sci..

[13] Zhang Y., Wang J. (2020). Targeting uptake transporters for cancer imaging and treatment. Acta Pharm. Sin. B.

[14] Juhász C., Dwivedi S., Kamson D.O., Michelhaugh S.K., Mittal S. (2014). Comparison of amino acid positron emission tomographic radiotracers for molecular imaging of primary and metastatic brain tumors. Mol. Imaging.

[15] Alam S., Doherty E., Ortega-Prieto P., Arizanova J., Fets L. (2023). Membrane transporters in cell physiology, cancer metabolism and drug response. Dis. Model. Mech..

[16] Gadsby D.C. (2009). Ion channels versus ion pumps: The principal difference, in principle. Nat. Rev. Mol. Cell Biol..

[17] Huang Y., Anderle P., Bussey K.J., Barbacioru C., Shankavaram U., Dai Z., Reinhold W.C., Papp A., Weinstein J.N., Sadée W. (2004). Membrane transporters and channels: Role of the transportome in cancer chemosensitivity and chemoresistance. Cancer Res..

[18] Vasiliou V., Vasiliou K., Nebert D.W. (2009). Human ATP-binding cassette (ABC) transporter family. Hum. Genom..

[19] Becchetti A. (2011). Ion channels and transporters in cancer. 1. Ion channels and cell proliferation in cancer. Am. J. Physiol.-Cell Physiol..

[20] Drew D., Boudker O. (2016). Shared molecular mechanisms of membrane transporters. Annu. Rev. Biochem..

[21] Li J., Shaikh S.A., Enkavi G., Wen P.-C., Huang Z., Tajkhorshid E. (2013). Transient formation of water-conducting states in membrane transporters. Proc. Natl. Acad. Sci. USA.

[22] Kilbourn M.R. (2017). Small Molecule PET Tracers for Transporter Imaging. Semin. Nucl. Med..

[23] Meyer H.J., Wienke A., Surov A. (2019). Associations between GLUT expression and SUV values derived from FDG-PET in different tumors-A systematic review and meta analysis. PLoS ONE.

[24] Mamede M., Higashi T., Kitaichi M., Ishizu K., Ishimori T., Nakamoto Y., Yanagihara K., Li M., Tanaka F., Wada H. (2005). [^18^F]FDG uptake and PCNA, Glut-1, and Hexokinase-II expressions in cancers and inflammatory lesions of the lung. Neoplasia.

[25] Scafoglio C.R., Villegas B., Abdelhady G., Bailey S.T., Liu J., Shirali A.S., Wallace W.D., Magyar C.E., Grogan T.R., Elashoff D. (2018). Sodium-glucose transporter 2 is a diagnostic and therapeutic target for early-stage lung adenocarcinoma. Sci. Transl. Med..

[26] Scafoglio C., Hirayama B.A., Kepe V., Liu J., Ghezzi C., Satyamurthy N., Moatamed N.A., Huang J., Koepsell H., Barrio J.R. (2015). Functional expression of sodium-glucose transporters in cancer. Proc. Natl. Acad. Sci. USA.

[27] Youland R.S., Kitange G.J., Peterson T.E., Pafundi D.H., Ramiscal J.A., Pokorny J.L., Giannini C., Laack N.N., Parney I.F., Lowe V.J. (2013). The role of LAT1 in ^18^F-DOPA uptake in malignant gliomas. J. Neuro-Oncol..

[28] Law I., Albert N.L., Arbizu J., Boellaard R., Drzezga A., Galldiks N., la Fougère C., Langen K.-J., Lopci E., Lowe V. (2019). Joint EANM/EANO/RANO practice guidelines/SNMMI procedure standards for imaging of gliomas using PET with radiolabelled amino acids and [^18^F] FDG: Version 1.0. Eur. J. Nucl. Med. Mol. Imaging.

[29] Somme F., Bender L., Namer I.J., Noël G., Bund C. (2020). Usefulness of ^18^F-FDOPA PET for the management of primary brain tumors: A systematic review of the literature. Cancer Imaging.

[30] Okubo S., Zhen H.-N., Kawai N., Nishiyama Y., Haba R., Tamiya T. (2010). Correlation of L-methyl-^11^C-methionine (MET) uptake with L-type amino acid transporter 1 in human gliomas. J. Neuro-Oncol..

[31] Langen K.-J., Hamacher K., Weckesser M., Floeth F., Stoffels G., Bauer D., Coenen H.H., Pauleit D. (2006). O-(2-[^18^F] fluoroethyl)-L-tyrosine: Uptake mechanisms and clinical applications. Nucl. Med. Biol..

[32] Pauleit D., Stoffels G., Schaden W., Hamacher K., Bauer D., Tellmann L., Herzog H., Bröer S., Coenen H.H., Langen K.-J. (2005). PET with O-(2-^18^F-fluoroethyl)-L-tyrosine in peripheral tumors: First clinical results. J. Nucl. Med..

[33] Mittra E.S., Koglin N., Mosci C., Kumar M., Hoehne A., Keu K.V., Iagaru A.H., Mueller A., Berndt M., Bullich S. (2016). Pilot preclinical and clinical evaluation of (4S)-4-(3-[^18^F] fluoropropyl)-L-glutamate (^18^F-FSPG) for PET/CT imaging of intracranial malignancies. PLoS ONE.

[34] Van Hée V.F., Labar D., Dehon G., Grasso D., Grégoire V., Muccioli G.G., Frédérick R., Sonveaux P. (2017). Radiosynthesis and validation of (±)-[^18^F]-3-fluoro-2-hydroxypropionate ([^18^F]-FLac) as a PET tracer of lactate to monitor MCT1-dependent lactate uptake in tumors. Oncotarget.

[35] Farsad M. (2020). FDG PET/CT in the Staging of Lung Cancer. Curr. Radiopharm..

[36] de Geus-Oei L.F., Ruers T.J., Punt C.J., Leer J.W., Corstens F.H., Oyen W.J. (2006). FDG-PET in colorectal cancer. Cancer Imaging.

[37] Yoo K.H. (2022). Staging and response assessment of lymphoma: A brief review of the Lugano classification and the role of FDG-PET/CT. Blood Res..

[38] Klimek K., Chen X., Sasaki T., Groener D., Werner R.A., Higuchi T. (2024). PET imaging of sodium-glucose cotransporters (SGLTs): Unveiling metabolic dynamics in diabetes and oncology. Mol. Metab..

[39] Kogai T., Brent G.A. (2012). The sodium iodide symporter (NIS): Regulation and approaches to targeting for cancer therapeutics. Pharmacol. Ther..

[40] Gulec S.A., Kuker R.A., Goryawala M., Fernandez C., Perez R., Khan-Ghany A., Apaza A., Harja E., Harrell M. (2016). ^124^I PET/CT in patients with differentiated thyroid cancer: Clinical and quantitative image analysis. Thyroid.

[41] Ramos C.D., Zantut Wittmann D.E., Etchebehere E.C., Tambascia M.A., Silva C.A., Camargo E.E. (2002). Thyroid uptake and scintigraphy using 99mTc pertechnetate: Standardization in normal individuals. Sao Paulo Med. J..

[42] Wiriyasermkul P., Nagamori S., Tominaga H., Oriuchi N., Kaira K., Nakao H., Kitashoji T., Ohgaki R., Tanaka H., Endou H. (2012). Transport of 3-fluoro-L-α-methyl-tyrosine by tumor-upregulated L-type amino acid transporter 1: A cause of the tumor uptake in PET. J. Nucl. Med..

[43] Nobusawa A., Kim M., Kaira K., Miyashita G., Negishi A., Oriuchi N., Higuchi T., Tsushima Y., Kanai Y., Yokoo S. (2013). Diagnostic usefulness of ^18^F-FAMT PET and L-type amino acid transporter 1 (LAT1) expression in oral squamous cell carcinoma. Eur. J. Nucl. Med. Mol. Imaging.

[44] Müller A., Chiotellis A., Keller C., Ametamey S.M., Schibli R., Mu L., Krämer S.D. (2014). Imaging tumour ATB0,+ transport activity by PET with the cationic amino acid O-2 ((2-[^18^F] fluoroethyl) methyl-amino) ethyltyrosine. Mol. Imaging Biol..

[45] Bading J.R., Kan-Mitchell J., Conti P.S. (1996). System A amino acid transport in cultured human tumor cells: Implications for tumor imaging with PET. Nucl. Med. Biol..

[46] Sudo H., Tsuji A.B., Sugyo A., Okada M., Kato K., Zhang M.R., Saga T., Higashi T. (2018). Direct comparison of 2-amino [3-11C] isobutyric acid and 2-amino [^11^C] methyl-isobutyric acid uptake in eight lung cancer xenograft models. Int. J. Oncol..

[47] Scarpelli M.L., Healey D.R., Mehta S., Quarles C.C. (2022). Imaging glioblastoma with ^18^F-fluciclovine amino acid positron emission tomography. Front. Oncol..

[48] Parent E.E., Schuster D.M. (2018). Update on ^18^F-fluciclovine PET for prostate cancer imaging. J. Nucl. Med..

[49] Hassanein M., Hight M.R., Buck J.R., Tantawy M.N., Nickels M.L., Hoeksema M.D., Harris B.K., Boyd K., Massion P.P., Manning H.C. (2016). Preclinical Evaluation of 4-[^18^F]Fluoroglutamine PET to Assess ASCT2 Expression in Lung Cancer. Mol. Imaging Biol..

[50] Lin W., Wang C., Liu G., Bi C., Wang X., Zhou Q., Jin H. (2020). SLC7A11/xCT in cancer: Biological functions and therapeutic implications. Am. J. Cancer Res..

[51] Semenza G.L. (2008). Tumor metabolism: Cancer cells give and take lactate. J. Clin. Investig..

[52] Payen V.L., Mina E., Van Hée V.F., Porporato P.E., Sonveaux P. (2020). Monocarboxylate transporters in cancer. Mol. Metab..

[53] Inazu M. (2014). Choline transporter-like proteins CTLs/SLC44 family as a novel molecular target for cancer therapy. Biopharm. Drug Dispos..

[54] Saiki I., Yara M., Yamanaka T., Uchino H., Inazu M. (2020). Functional Expression of Choline Transporter-Like Protein 1 in LNCaP Prostate Cancer Cells: A Novel Molecular Target. Biomol. Ther..

[55] Ahmed E.E., Andrews J.R., Ahmed M.E., Haloi R., Rhodes L.A., Mahon M., Carter D.A., Wei N., Thorpe M.P., Johnson G.B. (2026). Predictive value of interim ^11^C-choline PET/CT for response to radium-223 in metastatic castration-resistant prostate cancer. EJNMMI Res..

[56] Schmilovitz-Weiss H., Boltin D., Groshar D., Domachevsky L., Rosenbaum E., Issa N., Sapoznikov B., Goren I., Issachar A., Cohen-Naftaly M. (2021). [^11^C] choline as a potential PET/CT biomarker of liver cirrhosis: A prospective pilot study. Dig. Liver Dis..

[57] Li T.-T., An J.-X., Xu J.-Y., Tuo B.-G. (2019). Overview of organic anion transporters and organic anion transporter polypeptides and their roles in the liver. World J. Clin. Cases.

[58] Kitao A., Matsui O., Yoneda N., Kozaka K., Kobayashi S., Koda W., Inoue D., Ogi T., Yoshida K., Gabata T. (2020). Gadoxetic acid-enhanced MR imaging for hepatocellular carcinoma: Molecular and genetic background. Eur. Radiol..

[59] Peng F. (2022). Recent Advances in Cancer Imaging with (64)CuCl(2) PET/CT. Nucl. Med. Mol. Imaging.

[60] Capriotti G., Piccardo A., Giovannelli E., Signore A. (2022). Targeting copper in cancer imaging and therapy: A new theragnostic agent. J. Clin. Med..

[61] Warburg O. (1956). On the origin of cancer cells. Science.

[62] Koppenol W.H., Bounds P.L., Dang C.V. (2011). Otto Warburg’s contributions to current concepts of cancer metabolism. Nat. Rev. Cancer.

[63] Pliszka M., Szablewski L. (2021). Glucose transporters as a target for anticancer therapy. Cancers.

[64] Kawada K., Iwamoto M., Sakai Y. (2016). Mechanisms underlying (18)F-fluorodeoxyglucose accumulation in colorectal cancer. World J. Radiol..

[65] Wuest M., Hamann I., Bouvet V., Glubrecht D., Marshall A., Trayner B., Soueidan O.-M., Krys D., Wagner M., Cheeseman C. (2018). Molecular imaging of GLUT1 and GLUT5 in breast cancer: A multitracer positron emission tomography imaging study in mice. Mol. Pharmacol..

[66] El-Chemaly S., Malide D., Yao J., Nathan S.D., Rosas I.O., Gahl W.A., Moss J., Gochuico B.R. (2013). Glucose transporter-1 distribution in fibrotic lung disease: Association with [^18^F]-2-fluoro-2-deoxyglucose-PET scan uptake, inflammation, and neovascularization. Chest.

[67] Boellaard R., Delgado-Bolton R., Oyen W.J., Giammarile F., Tatsch K., Eschner W., Verzijlbergen F.J., Barrington S.F., Pike L.C., Weber W.A. (2015). FDG PET/CT: EANM procedure guidelines for tumour imaging: Version 2.0. Eur. J. Nucl. Med. Mol. Imaging.

[68] Sano R., Shinozaki Y., Ohta T. (2020). Sodium-glucose cotransporters: Functional properties and pharmaceutical potential. J. Diabetes Investig..

[69] Wright E.M., Loo D.D., Hirayama B.A. (2011). Biology of human sodium glucose transporters. Physiol. Rev..

[70] Jadvar H., Alavi A., Gambhir S.S. (2009). ^18^F-FDG uptake in lung, breast, and colon cancers: Molecular biology correlates and disease characterization. J. Nucl. Med..

[71] Basu S., Chen W., Tchou J., Mavi A., Cermik T., Czerniecki B., Schnall M., Alavi A. (2008). Comparison of triple-negative and estrogen receptor-positive/progesterone receptor-positive/HER2-negative breast carcinoma using quantitative fluorine-18 fluorodeoxyglucose/positron emission tomography imaging parameters: A potentially useful method for disease characterization. Cancer Interdiscip. Int. J. Am. Cancer Soc..

[72] Rahman W.T., Wale D.J., Viglianti B.L., Townsend D.M., Manganaro M.S., Gross M.D., Wong K.K., Rubello D. (2019). The impact of infection and inflammation in oncologic (18)F-FDG PET/CT imaging. Biomed. Pharmacother..

[73] Pu Y., Wang C., Zhao S., Xie R., Zhao L., Li K., Yang C., Zhang R., Tian Y., Tan L. (2021). The clinical application of ^18^F-FDG PET/CT in pancreatic cancer: A narrative review. Transl. Cancer Res..

[74] Wright E.M. (2020). SGLT2 and cancer. Pflüg. Arch.-Eur. J. Physiol..

[75] Kepe V., Scafoglio C., Liu J., Yong W.H., Bergsneider M., Huang S.-C., Barrio J.R., Wright E.M. (2018). Positron emission tomography of sodium glucose cotransport activity in high grade astrocytomas. J. Neuro-Oncol..

[76] Geist B.K., Ramirez J.C., Binder P., Einspieler H., Ibeschitz H., Langsteger W., Nics L., Rausch I., Diemling M., Sohlberg A. (2024). In vivo assessment of safety, biodistribution, and radiation dosimetry of the [^18^F]Me4FDG PET-radiotracer in adults. EJNMMI Res..

[77] Shikano N., Kawai K. (2020). Radiolabeled molecular imaging agents for amino acid transport in tumors. Bull. Ibaraki Prefect. Univ. Health Sci..

[78] Hushmandi K., Einollahi B., Saadat S.H., Lee E.H.C., Farani M.R., Okina E., Huh Y.S., Nabavi N., Salimimoghadam S., Kumar A.P. (2024). Amino acid transporters within the solute carrier superfamily: Underappreciated proteins and novel opportunities for cancer therapy. Mol. Metab..

[79] Häfliger P., Charles R.P. (2019). The L-Type Amino Acid Transporter LAT1-An Emerging Target in Cancer. Int. J. Mol. Sci..

[80] Yan R., Zhao X., Lei J., Zhou Q. (2019). Structure of the human LAT1-4F2hc heteromeric amino acid transporter complex. Nature.

[81] Scalise M., Galluccio M., Console L., Pochini L., Indiveri C. (2018). The Human SLC7A5 (LAT1): The Intriguing Histidine/Large Neutral Amino Acid Transporter and Its Relevance to Human Health. Front. Chem..

[82] Lopes C., Pereira C., Medeiros R. (2021). ASCT2 and LAT1 Contribution to the Hallmarks of Cancer: From a Molecular Perspective to Clinical Translation. Cancers.

[83] Cormerais Y., Giuliano S., LeFloch R., Front B., Durivault J., Tambutté E., Massard P.-A., de la Ballina L.R., Endou H., Wempe M.F. (2016). Genetic disruption of the multifunctional CD98/LAT1 complex demonstrates the key role of essential amino acid transport in the control of mTORC1 and tumor growth. Cancer Res..

[84] Wyant G.A., Abu-Remaileh M., Wolfson R.L., Chen W.W., Freinkman E., Danai L.V., Vander Heiden M.G., Sabatini D.M. (2017). mTORC1 Activator SLC38A9 Is Required to Efflux Essential Amino Acids from Lysosomes and Use Protein as a Nutrient. Cell.

[85] Koppula P., Zhuang L., Gan B. (2021). Cystine transporter SLC7A11/xCT in cancer: Ferroptosis, nutrient dependency, and cancer therapy. Protein Cell.

[86] Wu J., Shen Z.X., Yang W. (2020). Redox Mechanism in Na-Ion Battery Cathodes Probed by Advanced Soft X-Ray Spectroscopy. Front. Chem..

[87] Langen K.-J., Bröer S. (2004). Molecular transport mechanisms of radiolabeled amino acids for PET and SPECT. J. Nucl. Med..

[88] Harding H.P., Zhang Y., Zeng H., Novoa I., Lu P.D., Calfon M., Sadri N., Yun C., Popko B., Paules R. (2003). An integrated stress response regulates amino acid metabolism and resistance to oxidative stress. Mol. Cell.

[89] Wester H.J., Herz M., Weber W., Heiss P., Senekowitsch-Schmidtke R., Schwaiger M., Stöcklin G. (1999). Synthesis and radiopharmacology of O-(2-[^18^F] fluoroethyl)-L-tyrosine for tumor imaging. J. Nucl. Med..

[90] Kracht L.W., Miletic H., Busch S., Jacobs A.H., Voges J., Hoevels M., Klein J.C., Herholz K., Heiss W.-D. (2004). Delineation of brain tumor extent with [^11^C] L-methionine positron emission tomography: Local comparison with stereotactic histopathology. Clin. Cancer Res..

[91] Barollo S., Bertazza L., Watutantrige-Fernando S., Censi S., Cavedon E., Galuppini F., Pennelli G., Fassina A., Citton M., Rubin B. (2016). Overexpression of L-type amino acid transporter 1 (LAT1) and 2 (LAT2): Novel markers of neuroendocrine tumors. PLoS ONE.

[92] Guedj E., Varrone A., Boellaard R., Albert N.L., Barthel H., van Berckel B., Brendel M., Cecchin D., Ekmekcioglu O., Garibotto V. (2022). EANM procedure guidelines for brain PET imaging using [^18^F] FDG, version 3. Eur. J. Nucl. Med. Mol. Imaging.

[93] Arbizu J., Morbelli S., Minoshima S., Barthel H., Kuo P., Van Weehaeghe D., Horner N., Colletti P.M., Guedj E. (2025). SNMMI procedure standard/EANM practice guideline for brain [^18^F] FDG PET imaging, version 2.0. J. Nucl. Med..

[94] Salber D., Stoffels G., Pauleit D., Oros-Peusquens A.-M., Shah N.J., Klauth P., Hamacher K., Coenen H.H., Langen K.-J. (2007). Differential uptake of O-(2-^18^F-fluoroethyl)-L-tyrosine, L-3H-methionine, and 3H-deoxyglucose in brain abscesses. J. Nucl. Med..

[95] Floeth F.W., Pauleit D., Sabel M., Reifenberger G., Stoffels G., Stummer W., Rommel F., Hamacher K., Langen K.-J. (2006). ^18^F-FET PET differentiation of ring-enhancing brain lesions. J. Nucl. Med..

[96] Wongthai P., Hagiwara K., Miyoshi Y., Wiriyasermkul P., Wei L., Ohgaki R., Kato I., Hamase K., Nagamori S., Kanai Y. (2015). Boronophenylalanine, a boron delivery agent for boron neutron capture therapy, is transported by ATB^0,+^, LAT1 and LAT2. Cancer Sci..

[97] Hanaoka K., Watabe T., Naka S., Kanai Y., Ikeda H., Horitsugi G., Kato H., Isohashi K., Shimosegawa E., Hatazawa J. (2014). FBPA PET in boron neutron capture therapy for cancer: Prediction of (10)B concentration in the tumor and normal tissue in a rat xenograft model. EJNMMI Res..

[98] Lin K.H., Chen Y.W., Wang L.W., Wang Y.F., Hu L.H., Ting C.H., Lee T.H., Lee J.C., Peng N.J. (2024). Prognostic assessment of (18)F-boronophenylalanine positron emission tomography (BPA-PET) in salvage boron neutron capture therapy for malignant brain tumors. Quant. Imaging Med. Surg..

[99] Yamaguchi A., Hanaoka H., Fujisawa Y., Zhao S., Suzue K., Morita A., Tominaga H., Higuchi T., Hisaeda H., Tsushima Y. (2015). Differentiation of malignant tumours from granulomas by using dynamic [^18^F]-fluoro-L-α-methyltyrosine positron emission tomography. EJNMMI Res..

[100] Ikotun O.F., Marquez B.V., Huang C., Masuko K., Daiji M., Masuko T., McConathy J., Lapi S.E. (2013). Imaging the L-type amino acid transporter-1 (LAT1) with Zr-89 immunoPET. PLoS ONE.

[101] Vettermann F.J., Diekmann C., Weidner L., Unterrainer M., Suchorska B., Ruf V., Dorostkar M., Wenter V., Herms J., Tonn J.-C. (2021). L-type amino acid transporter (LAT) 1 expression in ^18^F-FET-negative gliomas. EJNMMI Res..

[102] Wei L., Tominaga H., Ohgaki R., Wiriyasermkul P., Hagiwara K., Okuda S., Kaira K., Oriuchi N., Nagamori S., Kanai Y. (2016). Specific transport of 3-fluoro-l-α-methyl-tyrosine by LAT1 explains its specificity to malignant tumors in imaging. Cancer Sci..

[103] Achmad A., Hanaoka H., Holik H.A., Endo K., Tsushima Y., Kartamihardja A.H.S. (2025). LAT1-specific PET radiotracers: Development and clinical experiences of a new class of cancer-specific radiopharmaceuticals. Theranostics.

[104] Kondo N., Hirano F., Kanai Y., Suzuki K., Miyazaki A., Temma T. (2026). A LAT1-selective PET tracer, 5-[^18^F]F-αMe-3BPA, as a companion to its structurally matched ^10^B analog in boron neutron capture therapy. Eur. J. Nucl. Med. Mol. Imaging.

[105] Rainu S.K., Ramachandran R.G., Parameswaran S., Krishnakumar S., Singh N. (2023). Advancements in Intraoperative Near-Infrared Fluorescence Imaging for Accurate Tumor Resection: A Promising Technique for Improved Surgical Outcomes and Patient Survival. ACS Biomater. Sci. Eng..

[106] Li K., Yang J., Lian H., Tian Z., Li C., Gao N., Guo Z. (2024). Cutting-edge insights: Near-infrared imaging for surgical margin assessment in head and neck tumor resection: A systematic review and meta-analysis. Quant. Imaging Med. Surg..

[107] Zhang W., Xu J., Dong L., Wang Y., Wu Y. (2026). A SLC7A5-Specific Near-Infrared Fluorescent Probe for Cancer-Targeted Imaging Applications. J. Fluoresc..

[108] Li S., Lin Z., Chen H., Luo Q., Han S., Huang K., Chen R., Zhan Y., Chen B., Yao H. (2024). Synthesis and Application of a Near-Infrared Light-Emitting Fluorescent Probe for Specific Imaging of Cancer Cells with High Sensitivity and Selectivity. Drug Des. Dev. Ther..

[109] Lu H., Xuan Y., Sun H., Miao Z., Zhan Y., Zhang Z., Zhang Q. (2025). One-pot synthesis of ultra-bright water-soluble gold nanoclusters for LAT1-targeted cancer cell imaging. Nanoscale.

[110] Isuri R.K., Hart M.C., Adusei-Poku S., Dudek G.C., Delikatny E.J., Popov A.V. (2026). Low molecular weight 4,4′-quinocyanines for in vivo NIR-II fluorescence imaging. npj Imaging.

[111] Wang J., Ye C., Chen C., Xiong H., Xie B., Zhou J., Chen Y., Zheng S., Wang L. (2017). Glucose transporter GLUT1 expression and clinical outcome in solid tumors: A systematic review and meta-analysis. Oncotarget.

[112] Rudlowski C., Moser M., Becker A.J., Rath W., Buttner R., Schroder W., Schurmann A. (2004). GLUT1 mRNA and protein expression in ovarian borderline tumors and cancer. Oncology.

[113] Vasudevan S., Mehta A., Sharma S.K., Sharma A. (2022). Expression of glucose transporter 1 (SLC2A1)—Clinicopathological associations and survival in an Indian cohort of colorectal cancer patients. J. Cancer Res. Ther..

[114] Fan R., Hou W.J., Zhao Y.J., Liu S.L., Qiu X.S., Wang E.H., Wu G.P. (2016). Overexpression of HPV16 E6/E7 mediated HIF-1α upregulation of GLUT1 expression in lung cancer cells. Tumour Biol..

[115] Reyna-Hernández M.A., Alarcón-Romero L.D.C., Ortiz-Ortiz J., Illades-Aguiar B., Jiménez-López M.A., Ocampo-Bárcenas A., Morrugares-Ixtepan M.O., Torres-Rojas F.I. (2022). GLUT1, LDHA, and MCT4 Expression Is Deregulated in Cervical Cancer and Precursor Lesions. J. Histochem. Cytochem..

[116] Heydarzadeh S., Moshtaghie A.A., Daneshpoor M., Hedayati M. (2020). Regulators of glucose uptake in thyroid cancer cell lines. Cell Commun. Signal.

[117] Macheda M.L., Rogers S., Best J.D. (2005). Molecular and cellular regulation of glucose transporter (GLUT) proteins in cancer. J. Cell. Physiol..

[118] Tsai T.H., Yang C.C., Kou T.C., Yang C.E., Dai J.Z., Chen C.L., Lin C.W. (2021). Overexpression of GLUT3 promotes metastasis of triple-negative breast cancer by modulating the inflammatory tumor microenvironment. J. Cell. Physiol..

[119] Masin M., Vazquez J., Rossi S., Groeneveld S., Samson N., Schwalie P.C., Deplancke B., Frawley L.E., Gouttenoire J., Moradpour D. (2014). GLUT3 is induced during epithelial-mesenchymal transition and promotes tumor cell proliferation in non-small cell lung cancer. Cancer Metab..

[120] Kim E., Jung S., Park W.S., Lee J.H., Shin R., Heo S.C., Choe E.K., Lee J.H., Kim K., Chai Y.J. (2019). Upregulation of SLC2A3 gene and prognosis in colorectal carcinoma: Analysis of TCGA data. BMC Cancer.

[121] Casneuf V.F., Fonteyne P., Van Damme N., Demetter P., Pauwels P., de Hemptinne B., De Vos M., Van de Wiele C., Peeters M. (2008). Expression of SGLT1, Bcl-2 and p53 in primary pancreatic cancer related to survival. Cancer Investig..

[122] Lai B., Xiao Y., Pu H., Cao Q., Jing H., Liu X. (2012). Overexpression of SGLT1 is correlated with tumor development and poor prognosis of ovarian carcinoma. Arch. Gynecol. Obstet..

[123] Gupta N., Miyauchi S., Martindale R.G., Herdman A.V., Podolsky R., Miyake K., Mager S., Prasad P.D., Ganapathy M.E., Ganapathy V. (2005). Upregulation of the amino acid transporter ATB^0,+^ (SLC6A14) in colorectal cancer and metastasis in humans. Biochim. Biophys. Acta.

[124] Luo Q., Yang B., Tao W., Li J., Kou L., Lian H., Che X., He Z., Sun J. (2017). ATB^0,+^ transporter-mediated targeting delivery to human lung cancer cells via aspartate-modified docetaxel-loading stealth liposomes. Biomater. Sci..

[125] Kou L., Huang H., Lin X., Jiang X., Wang Y., Luo Q., Sun J., Yao Q., Ganapathy V., Chen R. (2020). Endocytosis of ATB^0,+^(SLC6A14)-targeted liposomes for drug delivery and its therapeutic application for pancreatic cancer. Expert. Opin. Drug Deliv..

[126] Luo Q., Gong P., Sun M., Kou L., Ganapathy V., Jing Y., He Z., Sun J. (2016). Transporter occluded-state conformation-induced endocytosis: Amino acid transporter ATB^0,+^-mediated tumor targeting of liposomes for docetaxel delivery for hepatocarcinoma therapy. J. Control Release.

[127] Böhme-Schäfer I., Lörentz S., Bosserhoff A.K. (2022). Role of Amino Acid Transporter SNAT1/SLC38A1 in Human Melanoma. Cancers.

[128] Wang K., Cao F., Fang W., Hu Y., Chen Y., Ding H., Yu G. (2013). Activation of SNAT1/SLC38A1 in human breast cancer: Correlation with p-Akt overexpression. BMC Cancer.

[129] Baczewska M., Supruniuk E., Bojczuk K., Guzik P., Milewska P., Konończuk K., Dobroch J., Chabowski A., Knapp P. (2022). Energy Substrate Transporters in High-Grade Ovarian Cancer: Gene Expression and Clinical Implications. Int. J. Mol. Sci..

[130] Wang M., Liu Y., Fang W., Liu K., Jiao X., Wang Z., Wang J., Zang Y.S. (2017). Increased SNAT1 is a marker of human osteosarcoma and potential therapeutic target. Oncotarget.

[131] Bröer A., Gauthier-Coles G., Rahimi F., van Geldermalsen M., Dorsch D., Wegener A., Holst J., Bröer S. (2019). Ablation of the ASCT2 (SLC1A5) gene encoding a neutral amino acid transporter reveals transporter plasticity and redundancy in cancer cells. J. Biol. Chem..

[132] Morotti M., Zois C.E., El-Ansari R., Craze M.L., Rakha E.A., Fan S.J., Valli A., Haider S., Goberdhan D.C.I., Green A.R. (2021). Increased expression of glutamine transporter SNAT2/SLC38A2 promotes glutamine dependence and oxidative stress resistance, and is associated with worse prognosis in triple-negative breast cancer. Br. J. Cancer.

[133] Liu Y., Zhao T., Li Z., Wang L., Yuan S., Sun L. (2018). The role of ASCT2 in cancer: A review. Eur. J. Pharmacol..

[134] Ji X., Qian J., Rahman S.M.J., Siska P.J., Zou Y., Harris B.K., Hoeksema M.D., Trenary I.A., Heidi C., Eisenberg R. (2018). xCT (SLC7A11)-mediated metabolic reprogramming promotes non-small cell lung cancer progression. Oncogene.

[135] Guo W., Zhao Y., Zhang Z., Tan N., Zhao F., Ge C., Liang L., Jia D., Chen T., Yao M. (2011). Disruption of xCT inhibits cell growth via the ROS/autophagy pathway in hepatocellular carcinoma. Cancer Lett..

[136] Dai L., Cao Y., Chen Y., Parsons C., Qin Z. (2014). Targeting xCT, a cystine-glutamate transporter induces apoptosis and tumor regression for KSHV/HIV-associated lymphoma. J. Hematol. Oncol..

[137] Puri S., Juvale K. (2020). Monocarboxylate transporter 1 and 4 inhibitors as potential therapeutics for treating solid tumours: A review with structure-activity relationship insights. Eur. J. Med. Chem..

[138] Reuss A.M., Groos D., Ghoochani A., Buchfelder M., Savaskan N. (2021). MCT4 Promotes Tumor Malignancy in F98 Glioma Cells. J. Oncol..

[139] Yuan C., Zhang J., Lou J., Wang S., Jiang Y., Wu F., Wang S. (2021). Comprehensive Analysis of Monocarboxylate Transporter 4 (MCT4) expression in breast cancer prognosis and immune infiltration via integrated bioinformatics analysis. Bioengineered.

[140] Lee J.Y., Lee I., Chang W.J., Ahn S.M., Lim S.H., Kim H.S., Yoo K.H., Jung K.S., Song H.N., Cho J.H. (2016). MCT4 as a potential therapeutic target for metastatic gastric cancer with peritoneal carcinomatosis. Oncotarget.

[141] Duan X., Xie Y., Yu J., Hu X., Liu Z., Li N., Qin J., Lan L., Yuan M., Pan Z. (2022). MCT4/Lactate Promotes PD-L1 Glycosylation in Triple-Negative Breast Cancer Cells. J. Oncol..

[142] Penet M.F., Shah T., Bharti S., Krishnamachary B., Artemov D., Mironchik Y., Wildes F., Maitra A., Bhujwalla Z.M. (2015). Metabolic imaging of pancreatic ductal adenocarcinoma detects altered choline metabolism. Clin. Cancer Res..

[143] Hashimoto N., Nakajima K., Akashi Y., Kokubun K., Sugahara K., Katakura A., Matsuzaka K. (2025). Choline transporter-like protein 1 in tongue squamous cell carcinoma: Implications for cell proliferation and differentiation. Sci. Rep..

[144] Kim K.I., Jang S.J., Park J.H., Lee Y.J., Lee T.S., Woo K.S., Park H., Choe J.G., An G.I., Kang J.H. (2014). Detection of increased ^64^Cu uptake by human copper transporter 1 gene overexpression using PET with ^64^CuCl_2_ in human breast cancer xenograft model. J. Nucl. Med..

[145] Chen H.H., Yan J.J., Chen W.C., Kuo M.T., Lai Y.H., Lai W.W., Liu H.S., Su W.C. (2012). Predictive and prognostic value of human copper transporter 1 (hCtr1) in patients with stage III non-small-cell lung cancer receiving first-line platinum-based doublet chemotherapy. Lung Cancer.

[146] Qin C., Liu H., Chen K., Hu X., Ma X., Lan X., Zhang Y., Cheng Z. (2014). Theranostics of malignant melanoma with ^64^CuCl_2_. J. Nucl. Med..

